# Respiratory and cardiac motion correction in dual gated PET/MR imaging

**DOI:** 10.1186/2197-7364-2-S1-A43

**Published:** 2015-05-18

**Authors:** Hadi Fayad, Florian Monnier, Freedy Odille, Jacques Felblinger, Frederic Lamare, Dimitris Visvikis

**Affiliations:** LaTIM, INSERM, UMR 1101, Brest, France; INSERM U947, University of Nancy, Nancy, France; INCIA, UMR5287, CNRS, CHU Bordeaux, Bordeaux, France

Respiratory and cardiac motion in PET/MR imaging leads to reduced quantitative and qualitative image accuracy. Correction methodologies involve the use of double gated acquisitions which lead to low signal-to-noise ratio (SNR) and to issues concerning the combination of cardiac and respiratory frames. The objective of this work is to use a generalized reconstruction by inversion of coupled systems (GRICS) approach, previously used for PET/MR respiratory motion correction, combined with a cardiac phase signal and a reconstruction incorporated PET motion correction approach in order to reconstruct motion free images from dual gated PET acquisitions. The GRICS method consists of formulating parallel MRI in the presence of patient motion as a coupled inverse problem. Its resolution, using a fixed-point method, allows the reconstructed image to be improved using a motion model constructed from the raw MR data and two respiratory belts. GRICS obtained respiratory displacements are interpolated using the cardiac phase derived from an ECG to model simultaneous cardiac and respiratory motion. Three different volunteer datasets (4DMR acquisitions) were used for evaluation. GATE was used to simulate 4DPET datasets corresponding to the acquired 4DMR images. Simulated data were subsequently binned using 16 cardiac phases (M1) vs diastole only (M2), in combination with 8 respiratory amplitude gates. Respiratory and cardiac motion corrected PET images using either M1 or M2 were compared to respiratory only corrected images and evaluated in terms of SNR and contrast improvement. Significant visual improvements were obtained when correcting simultaneously for respiratory and cardiac motion (using 16 cardiac phase or diastole only) compared to respiratory motion only compensation. Results were confirmed by an associated increased SNR and contrast. Results indicate that using GRICS is an efficient tool for respiratory and cardiac motion correction in dual gated PET/MR imaging.

